# The advent of medical artificial intelligence: lessons from the Japanese approach

**DOI:** 10.1186/s40560-020-00452-5

**Published:** 2020-05-18

**Authors:** Euma Ishii, Daniel K. Ebner, Satoshi Kimura, Louis Agha-Mir-Salim, Ryo Uchimido, Leo A. Celi

**Affiliations:** 1 Department of Global Health Promotion, Tokyo Medical and Dental University, 1 Chome-5-45 Yushima, Bunkyo City, Tokyo, 113-8510 Japan; 2 Department of Intensive Care Medicine, Tokyo Medical and Dental University, 1 Chome-5-45 Yushima, Bunkyo City, Tokyo, 113-8510 Japan; 3 Institute for Medical Engineering and Science, Massachusetts Institute of Technology, 77 Massachusetts Avenue, E25-505, Cambridge, MA 02142 USA; 4 Alpert Medical School of Brown University, 222 Richmond St, Providence, RI 02906 USA; 5 Department of Anesthesiology and Resuscitation, Okayama University Hospital, 2-5-1 Shikata-cho, Kita-ku, Okayama, 700-8558 Japan; 6 Faculty of Medicine, University of Southampton, University Road, Southampton, SO17 1BJ UK; 7 Beth Israel Deaconess Medical Center, 330 Brookline Avenue, Boston, MA 02215 USA

**Keywords:** AI, Next Generation Medical Foundation Law, My Number System, Society 5.0, Big Data, Partnerships

## Abstract

Artificial intelligence or AI has been heralded as the most transformative technology in healthcare, including critical care medicine. Globally, healthcare specialists and health ministries are being pressured to create and implement a roadmap to incorporate applications of AI into care delivery. To date, the majority of Japan’s approach to AI has been anchored in industry, and the challenges that have occurred therein offer important lessons for nations developing new AI strategies. Notably, the demand for an AI-literate workforce has outpaced training programs and knowledge. This is particularly observable within medicine, where clinicians may be unfamiliar with the technology. National policy and private sector involvement have shown promise in developing both workforce and AI applications in healthcare. In combination with Japan’s unique national healthcare system and aggregable healthcare and socioeconomic data, Japan has a rich opportunity to lead in the field of medical AI.

## Introduction

Developing global artificial intelligence (AI) talent is a challenge, with a 2018 review presenting a geographic maldistribution of identified experts: half in the US (10,295), then China (2525), and the UK (1475) [1]. Only 805 were from Japan (3.6%), despite Japan’s large population in comparison with European nations, highlighting a challenge in talent development. A 2017 review by Nikkei and Elsevier reported that only the University of Tokyo was within the top 100 organizations with frequently cited research papers in the field of AI [2]. The delay in rearing AI talent in Japan has further challenges when AI is integrated with other complex industries such as medicine and healthcare. To address these challenges, Japan’s current public and private sector strategy is to create an environment that generates sufficient AI talent. Japan’s efforts may serve as a reference point for policy enactment. This paper provides an overview of Japan’s approach to developing local AI talent, and the resources devoted therein, in order to inform future local and international strategies in medicine.

## Japan's national strategy for developing AI leaders and technology

To address the relative shortage in AI expertise, Japan launched the AI Technology Strategy Council in 2016, naming AI as a key technological foundation for its 5th Science and Technology Basic Plan for the future of Japanese society “Society 5.0” [3]. Specifically, the strategy focuses on productivity, health, and mobility [4], with corresponding investments in research and development (R&D), talent recruitment, public data, and start-up companies. The council’s strategy was released in 2017 along with a roadmap divided into three phases: (1) the development and application of AI within various domains, (2) the public use of data and AI across those domains, and (3) the creation of ecosystems that integrate domains together [5]. Japan’s AI market is estimated to grow from JPY 3.7 trillion (USD 35 billion) in 2015 to JPY 87 trillion (USD 821 billion) by 2030 [6, 7], and the policy aims to address a shortage of AI engineers by capitalizing on pre-existing R&D collaborations between industry, academia, and government.

This physical and social infrastructure offers further benefits within healthcare. Through the recent enactment of the Next Generation Medical Foundation Law [8] combined with the My Number System [9], Japan has an opportunity to curate its 125 million citizen’s data into a central anonymized repository [10]*.* This would represent one of the largest central repositories of curated information to date and a substantial asset in the development of a national and international medical AI strategy. Non-socialized healthcare systems such as the US cannot replicate this, while other socialized nations such as the UK have similar infrastructure yet smaller overall population. Thus, there is a significant and unique opportunity for Japan to lead the world in the advent of medical AI [10].

To this end, the Japanese government has formed collaborations with industry and academic institutions to develop ten AI-enhanced hospitals by fiscal year 2022 [11]. It further mandated a beginner AI/data science course for approximately 40% of college graduates [12], 48% within STEM areas, and half of those within the health sciences [13]. Moreover, private efforts to promote medical AI applications are expanding; Preferred Networks, Inc. [14] in partnership with the Japan AI Medical Society [15] created a free online course on the foundations of medical AI [16]. Further efforts by the public and private sector are expected and are outlined in Table 1 below [17].

**Table 1:** Initiatives by the Ministry of Health, Labor, and Welfare (MHLW) to promote AI in medicine

Field	Initiatives spearheaded by the MHLW
**Genomic medicine**	● Establishment of the Center for Cancer Genome Information Management within the National Cancer Center and aggregate genome information ● Creation of a central information database from which clinical and genetic information would be analyzed by the Cancer Genome Information Management Center
**Image diagnosis support**	● Creation of a diagnostic image database through a collaboration with various academic societies (Japanese Society of Pathology, the Japanese Society of Gastrointestinal Endoscopy, the Japanese Society of Radiology, and the Japanese Society of Ophthalmology, etc.) ● Implementation of guidelines through the Medical Practitioners Act and the Pharmaceuticals and Medical Devices Act
**Diagnostic and treatment support**	● Build an information infrastructure that covers a wide range of intractable diseases with research funding from the Japan Medical Research and Development Organization (AMED) ● Implementation of guidelines through the Medical Practitioners Act and the Pharmaceuticals and Medical Devices Act
**Drug development**	● Creation of a knowledge database to locate drug targets with the National Institute of Biomedical Innovation and Health and Nutrition (NIBIO) ● Matching pharmaceutical and IT companies with support from the National Institute of Biomedical Innovation and Health and Nutrition, RIKEN, and Kyoto University
**Nursing care and dementia**	● Provision of grants for the development of data collection and prediction tools for early detection and prevention of serious illnesses in nursing care
**Surgical support**	● Provision of grants for the standardization of the interface for interlinking surgical data

## AI for clinicians in Japan

Medical AI is still a nascent field worldwide. Leading vendors overseas include Dynamed [18], UpToDate [19], and VisualDx [20], which all provide evidence-based decision support technologies through smartphone and tablet deployable models. Japan’s super-aging society and low clinician to population ratio (2.4 clinicians per 1000) presents a unique healthcare ecosystem for R&D and trialing of novel AI technology [16]. Technological development within Japan to date appears to focus predominantly on automation of existing diagnostic modalities, with the opportunity for widespread clinic and hospital deployment.

In a study conducted by Osaka University, JST PRESTO, the University of Tokyo, and RIKEN, researchers developed a novel deep neural network called “MNet” to classify multiple neurological diseases using resting-state MEG signals, generating in particular high specificity [21]. Jo et al. suggest that the use of MNet as a classifier may improve neurological diagnoses and generate high specificity. In critical care, MEG-based diagnosis can be time-consuming and requires significant experience; thus, the application of AI to analyze MEG signals is expected to significantly reduce the burden on clinicians.

The use of AI within disease classification extends to other domains, such as oncology. The University of Tokyo, Shimadzu Corporation, and Juntendo University developed a predictive model that reduced misclassification rates of disease by approximately 50% in comparison with a single tumor marker [22]. Similarly, various institutes in Japan, Germany, the US, and Chile have worked together to enhance histology classification of breast tumors using subtle morphological differences of microenvironmental myoepithelial cell nuclei [23]. The deployment of these technologies in rural or resource-challenged areas of the country may maintain a high quality of care while reducing the need to transfer specimens across institutions. The applications of AI use in pathology may also promote the development of other telemedicine solutions.

Mental distress during or after admission to the ICU has been previously documented and described [24]. Keio University has developed the Project for Objective Measures Using Computational Psychiatry Technology (PROMPT), via a combination of biometrics, computer vision, and voice recognition to aid diagnosis for psychiatric conditions [25]. The project has also facilitated the collaboration between academia and industry partners such as Omron, Softbank, and Microsoft. The group also started the Understanding Psychiatric Illness through Natural Language Processing (UNDERPIN) project, employing natural language processing tools to discover characteristics of diseases based on linguistic information, opening new approaches for diagnosis and treatment [25]. The deployment of AI integrating social and medical data in all environments including the ICU may aid in developing tools to enhance personalized patient-physician interactions throughout the care process.

Development of these new algorithms and tools is only the first step toward innovation, as they must further be tested, verified, and approved. Japan has introduced two regulatory policies for this: First, approval under the “Act on Securing Quality, Efficacy and Safety of Products Including Pharmaceuticals and Medical Devices” is required for the use of AI software and devices as clinical decision support (CDS) tools, specifically for diagnosis and treatment [10, 26]. Second, in December 2018, the MHLW established that AI systems would be limited to diagnosis and treatment support, with any actions constituting a medical decision made exclusively by physicians [26]. The challenge of this approach is exemplified by the Computer-Aided Diagnosis (CAD) system, which employs an AI engine to detect common breast cancer patterns on mammography [27]. Within this framework, a system may aim to notify patients to schedule an appointment as soon as the system identifies a high likelihood of malignancy, but the requirement for human supervision may slow or wholly obviate this process.

## AI in healthcare systems

Collaborations between hospitals and industry are extending AI beyond the bedside to the system level. Fujitsu Ltd., for instance, has aimed to integrate AI in both administrative procedures as well as the management of patient data [28]. Allm Inc., an early success in Japan’s medical AI startup space, developed a platform for the field triage of patients with acute ischemic stroke [29]. The platform is a combined database of all regional stroke centers, evaluated according to their capability to provide endovascular or systemic thrombolytic treatment. Patients are triaged by using a clinically proven questionnaire, real-time traffic information, and nearest stroke center capabilities [30]. ALMEX Inc.’s “Sma-pa TERMINAL” provides an AI assistant to manage administrative tasks, including patient registration and billing, leveraging facial recognition for registration while synchronizing visit information to the patient’s mobile device [31]. Implementation of these tools may reduce the administrative burden at all levels of healthcare, freeing financial, and human resources.

Within the academic setting, researchers at the Tokyo Medical and Dental University, Keio University, Waseda University, and Tokyo Denki University are developing an algorithm to identify high-risk groups for domestic violence and abuse in Tokyo’s Adachi Ward, aiming to develop an AI-based system for prevention [32]. The system will integrate public health centers with patients and clinicians to facilitate risk assessment and early intervention.

Large social networking companies such as LINE have also entered the medical field to create clinically relevant platforms for individual users. In January 2019, LINE announced the launch of “LINE Healthcare,” a company established as a joint venture between LINE Corporation and Japanese medical web portal M3, Inc., to create online healthcare businesses [33]. The two companies expect to launch a new remote healthcare consulting service during 2019 and are considering the development of a new drug delivery service that utilizes M3’s existing pharmacist membership network. These partnerships across various industrial sectors promote the combination of behavioral data with health data, permitting access to health information via a tool already saturating the population.

## AI in critical care

The necessity of swift communication, rapid decision making, and constant information flow makes the ICU both a challenging and intriguing area for AI deployment. Difficulty in monitoring the physiological and pathophysiological mechanisms that sway patient conditions cause physicians to make critical decisions based on incomplete information under tight time constraints. Thus, the utilization of AI in the adaptation, extension, and integration of traditional decision-making tools has been explored for several decades through various attempts to alleviate physician burdens [34].

Specific areas that AI has been incorporated include severity scoring and mortality prediction, early detection and prediction of diseases, decision support in mechanical ventilation, and continuous data capture [35]. AI-supported interventions are aimed to decrease inter-clinician variability and have already shown promising results by outperforming traditional clinical practices [35]. In a study of a deep learning model used to predict cardiac arrest or acute respiratory failure from 1 to 6 h prior to onset resulted with the AI solution performing better, with a higher area under the receiver operating characteristic curve score values in comparison to two other conventional risk scores [36]. The AI solution improved predictions by 40% on average, outperforming other traditional warning scores such as area under the receiver operating characteristic (AUROC) [36]. However, although there is a high potential for AI in critical care, challenges of data security and patient privacy along with appropriate consent remain to be considered [35].

Japan is still in its primary stages of creating infrastructure that permits the collection of all available information into an environment for data utilization. In addition, physicians, data scientists, programmers, and engineers tend to work in isolation, heightening the barriers in multi-sectoral collaboration for appropriate information extraction, data analysis, and data interpretation [37]. The Japanese Society of Intensive Care Medicine (JSICM) and Massachusetts Institute of Technology’s (MIT) Critical Data team have partnered to combat these challenges by hosting annual “datathons” to bring professionals from various fields to collaborate and learn from one another while tackling critical care problems in the form of a hackathon [38, 39].

## Challenges and opportunities ahead

Much of the AI talent in Japan is concentrated in research institutes and industry [7]. However, there are a number of national and international programs that are helping to build a medical AI workforce [39]. The Mitou Foundation [40] selects gifted students under the age of 17 as part of a human resource discovery and development project under the jurisdiction of the Ministry of Economy, Trade and Industry [41]. Since 2000, the program provides mentoring from the Toyota Research Institute, among others, and has produced 1600 engineers, including DeNA founder Tomoko Minamiba and Ryo Morikawa of LINE [40]. Son Masayoshi of Softbank has also created a foundation to support the training of talented youth in various fields, including AI [41]. International collaborations between Kyoto Prefectural University of Medicine and Tokyo Medical and Dental University with MIT Critical Data also demonstrate efforts in promoting AI talent by fostering the collaboration of clinicians and data scientists [38, 39]. However, more work remains if the talent and leadership gaps between Japan and countries at the forefront of AI research are to be narrowed.

**Fig. 1:**
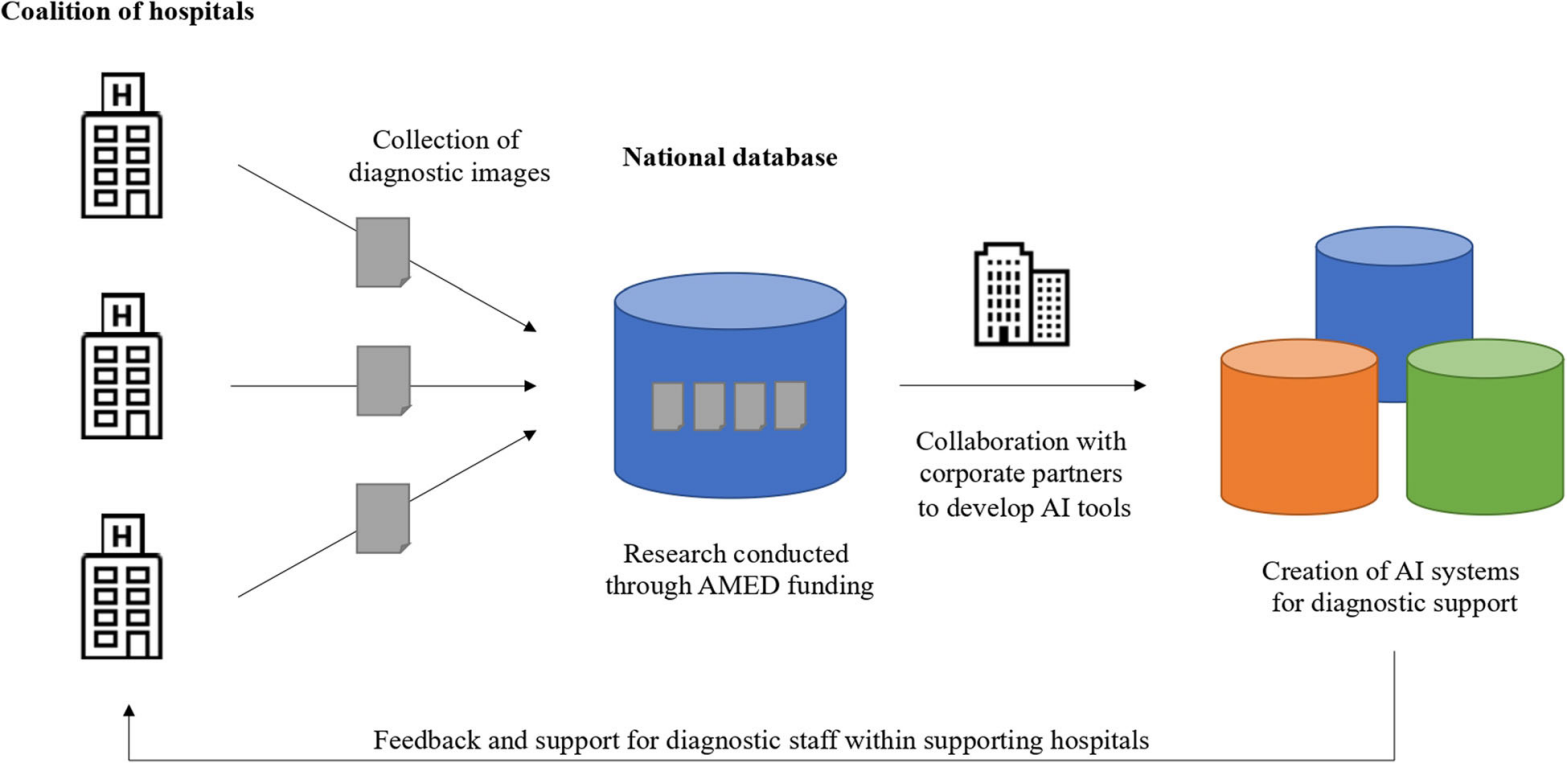
Example of a clinical diagnostic database to promote the development of supplementary AI tools in healthcare

Son Masayoshi publicly stated that Japan is an AI “*Koshinkoku*”—a nation left behind [42]. However, there exists a tremendous opportunity for the development and deployment of AI in fields including medicine in Japan, as ongoing research cited above demonstrates. Japan is uniquely situated in the developed world for medical algorithmic technology deployment, with a technologically savvy populace, well-developed healthcare system founded on universal coverage, and pre-existing academic, government, and industrial collaborative alignments. Critical to the development of a robust AI ecosystem is the availability of relevant, reliable data at scale, and the ongoing creation of shared clinical databases throughout Japan underscore this significant potential. If properly anonymized, made readily available to researchers, and paired with advancing educational initiatives, these datasets have a high potential in catalyzing a healthcare revolution in Japan (Fig. 1).

## Conclusion

The deployment of AI represents a unique challenge for every nation and nowhere more so than in the improvement of healthcare. Japan faces unique challenges and opportunities anchored in its unique social and technological infrastructure and will serve as a model for technology deployment in tackling common global problems including an aging population, with increasing rural and depopulated areas. Japan works today to develop a medical education system to create a workforce competent in the utilization, evaluation, and improvement of AI and to deploy thoughtful multidisciplinary and multilateral efforts to identify and disseminate best practices from patient-care to system-wide management. Through such an approach, it is uniquely situated to demonstrate to the world a well-designed roadmap of an AI-driven future of healthcare.

## Data Availability

Not applicable.

## Abbreviations

AI: Artificial intelligence
AUROC: Area under the receiver operating characteristic
CAD: Computer-aided diagnosis
CDS: Clinical decision support
EEG: Electroencephalography
JST: Japan Science and Technology Agency
MEG: Magnetoencephalography
PRESTO: Precursory Research for Embryonic Science and Technology
PROMPT: Project for Objective Measures Using Computational Psychiatry Technology
R&D: Research and development
STEM: Science, technology, engineering, and mathematics
UNDERPIN: Understanding Psychiatric Illness through Natural Language Processing
USD: United States Dollar
